# Impact of Postoperative Radiotherapy on Survival of Patients With *de novo* Stage IV Breast Cancer: A Population-Based Study From the SEER Database

**DOI:** 10.3389/fonc.2021.625628

**Published:** 2021-03-18

**Authors:** Jie Zhang, Shiping Luo, Zhaozhen Qiu, Yuxiang Lin, Chuangui Song

**Affiliations:** ^1^Department of Breast Surgery, Fujian Medical University Union Hospital, Fuzhou, China; ^2^Department of General Surgery, Fujian Medical University Union Hospital, Fuzhou, China; ^3^Breast Surgery Institute, Fujian Medical University, Fuzhou, China; ^4^Department of General Surgery, Second Affiliated Hospital of Fujian Medical University, Quanzhou, China

**Keywords:** stage IV, breast cancer, operation, radiotherapy, SEER

## Abstract

**Purpose:** In our study, we aimed to evaluate the role of postoperative radiotherapy for patents with *de novo* stage IV breast cancer.

**Patients and Methods:** Patients diagnosed with stage IV breast cancer from 2010 to 2016 were selected from the Surveillance, Epidemiology, and End Result (SEER) database. Those patients who received both chemotherapy and surgery and lived longer than 6 months were divided into radiotherapy and non-radiotherapy groups. Kaplan-Meier analysis and multivariate Cox proportional hazards models were used to estimate the survival outcomes before and after being 1:1 propensity score matched (PSM). Subgroup analyses stratified by age, subtype, status of distant metastasis, and surgery type were also performed.

**Results:** Among 1,935 patients, 52% (1006) underwent radiotherapy while the non-radiotherapy group contained 48% (929). After PSM, a total of 1,520 patients in two groups of 760 patients were enrolled in this analysis. Kaplan-Meier and the multivariate survival analysis demonstrated that the radiotherapy group presented with a better prognosis compared to the non-radiotherapy group (after PSM, BCSS: Hazard Ratio, 0.697; 95% confidence interval, 0.59–0.823; *P* < 0.001; OS: Hazard Ratio, 0.707; 95% confidence interval, 0.601–0.831; *P* < 0.001). Further subgroup analyses showed the Luminal subtype (HR+/HER2–), triple-negative breast cancer (TNBC), and bone-only metastasis patients presented with the most promising survival in the radiotherapy group.

**Conclusions:** Postoperative radiotherapy is associated with a significant survival advantages in BCSS and OS. It can be an optimal supplementary treatment for stage IV patients after surgery, especially for Luminal subtype, TNBC, and patients with a low metastatic burden.

## Introduction

*De novo* metastatic breast cancer (dnMBC), including synchronous metastasis of viscera, bone, or distant lymph nodes, when initially diagnosed is regarded as an incurable disease. Since the tumor has spread to organs other than the breast, systemic therapy, such as chemotherapy, endocrine therapy, or targeted therapy, are the mainstays of treatment for dnMBC. Local-regional treatment (LRT), including surgery or radiotherapy, were once considered as palliative treatment that would not affect the prognosis (1, 2). However, the findings of several randomized clinical trials brought growing debate over the local treatments of metastatic breast cancer (3–6). Two randomized clinical trials, TATA and MF07-01, that included surgery and radiotherapy in experimental groups, produced opposite conclusions. TATA did not agree with the local regional treatment as part of routine practice in stage IV patients since no positive result was observed. On the contrary, MF07-01 achieve statistically significant improvement of local regional treatment in a 40-month follow-up study. It should be noted that the median follow-up time was only 23 months in the TATA study but 40 months in the MF07-01 study. Insufficient follow-up time may also lead to erroneous assessment of potential survival differences in the TATA study. Although the results were contradictory, there was still a trend that could be found from MF07-01 that those patients who have a better prognosis or who have fewer metastasis sites were more likely to benefit from aggressive local treatments. This phenomenon was also demonstrated in some retrospective studies (7, 8). As the development of systemic treatment significantly improved the survival of advanced breast cancer patients, the clinical value of local treatment might need to be re-evaluated (9, 10).

Although surgery for stage IV breast cancer is still debated, a lot of studies have focused on it and demonstrated surgery to be effective in at least a selected population (11–16). Yet, evidence about the role of radiotherapy is rare. The conclusion of several retrospective studies indicate that radiation can be helpful for dnMBC patients. Both Le et al. and Elvire et al. demonstrated that exclusive radiotherapy (ERT) was associated with a significantly better OS in dnMBC patients than no-ERT (17, 18). These results indicated the potential efficacy of radiotherapy in dnMBC. Current discussions as to whether adding radiotherapy to surgery can bring additional effects to surgery alone are an active topic of debate. Only one study has focused on this area: Yi-jun Kim et al. evaluated the efficacy of adding radiotherapy to surgery in their study and concluded that combined treatment may increase the survival rate in dnMBC patients when compared with surgery alone (19). We feel that not considering the status of systemic treatment is a big limitation of their study. To make up for this deficiency, we conducted a study using the SEER database and analyzed the efficacy of postoperative radiotherapy in the patients who received both chemotherapy and surgery. We also used the propensity score matching (PSM) method to reduce the inherent selection bias of a retrospective study which may affect the accuracy of the results.

## Materials and Methods

### Patients

We conducted a retrospective, population-based cohort study to evaluate the role of postoperative radiation in treatment of *de novo* stage IV breast cancer patients. The SEER^*^Stat (version 8.3.4) database was searched to enroll the patients with an initial diagnosis of stage IV breast cancer who were recorded in the SEER program from 2010 to 2016. The prognosis varies greatly in different breast cancer subtypes which may also influence the evaluation of radiation. We selected the data of cases diagnosed from 2010 to 2016 because these data include the subtype details.

The pre-specified inclusion and exclusion criteria are as follows: All of the patients enrolled were initially diagnosed with stage IV breast cancer, survived at least 6 months, and had chemotherapy as well as surgery for treatment of their primary tumor and axillary lymph nodes. Unlike the previous study by Yi-jun Kim, we excluded those patients who did not receive chemotherapy or died before 6 months after diagnosis to ensure all of the enrolled patients were sensitive to systemic therapy and minimize the select bias by excluding those patients with uncontrolled disease who received radiotherapy as palliative treatment (19). No information on the radiation sites was provided by the SEER data. We excluded those patients who receive radiation of metastatic sites as palliative treatment as much as possible. Since radiation for the brain metastasis is most used besides radiation for the local regional area, those patients with brain metastases were excluded. Bone is another area that is recommended to receive radiation to relieve pain or strengthen stability when metastasis occurs. The patients with bone metastases were put into subgroups for independent analysis. The patients with primary tumor status T1-2N0 were also excluded. Hence, we could retain the patients with a high risk of local recurrence and ensure the radiotherapy was most probable used to reduce recurrence. In addition, those patients with T1-2N0 who received mastectomy would not be recommended to receive local regional radiotherapy, even those with early breast cancer. Therefore, it is more probable that radiotherapy was used as a palliative approach for distant sites.

The patients were divided into two groups; one that received radiotherapy after breast surgery and the other not (Radiation group and Non-radiation group). They were further categorized according to the metastatic location as bone-only metastasis, viscera metastasis, viscera + bone metastasis, and others (mainly including patients with distant lymph nodes metastasis). Demographic information including age, race, and marital status, and disease characteristics were compiled. Tumor status and nodal status were categorized according to the staging system of the AJCC 8th Edition. Histological grades were categorized into I, II, and III/IV. Types of surgery were categorized to mastectomy and BCS. Breast tumor subtypes were categorized into hormone receptor (HR)-positive and human epidermal growth factor receptor 2 (HER2)-negative, HR-positive and HER2-positive, HR-negative and HER2-positive, HR-negative and HER2-negative (also known as triple negative breast cancer, TNBC).

Overall survival (OS) was the primary end point of our study. It was defined as the time from diagnosis to death. The patients who were alive at the last time follow-up were censored. Breast cancer specific survival (BCSS) was the secondary end point.

### Statistical Analysis

We used the Chi-squared test to compare the demographic and clinical characteristics between the radiotherapy and non-radiotherapy groups in both the whole groups and 1:1 propensity score matched groups. The Kaplan-Meier analysis method computed the probabilistic differences in survival between the radiotherapy and non-radiotherapy groups. And the Cox proportional hazard regression models were used to assess the effects of survival between patients with stage IV breast cancer who was received radiotherapy vs. non-radiotherapy after adjusting for the confounding prognostic factors including age at diagnosis, race/ethnicity, marital status, the histological grades I-IV, T status (tumor size category), N status (lymph node status), the subtype of breast cancer, the status of distant metastasis, and the type of surgery. To compare the radiotherapy and non-radiotherapy groups for BCSS and OS we made a hierarchical analysis of four variables, including age, breast subtype, status of distant metastasis, and types of surgery. *P*-values < 0.05 were considered significant and all analyses were conducted using SPSS version 22.0 software (IBM SPSS Statistics, Chicago, IL, US). The psmatch2 module was used to perform propensity score matching (PSM)in Stata version 13.0 (20).

## Results

### Demographics and Clinical Characteristics of the Study Population

**Figure 1:**
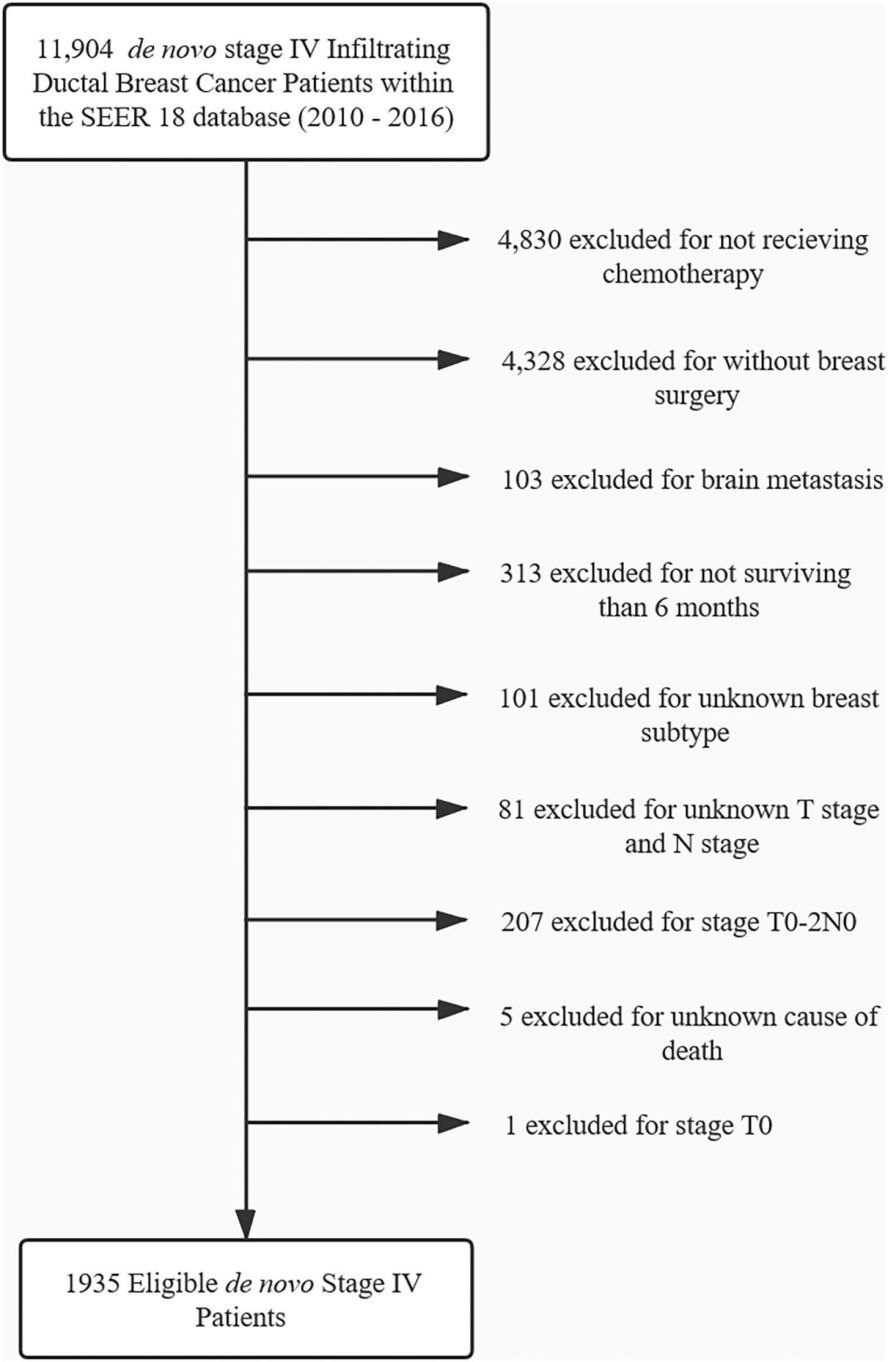
Consort diagram for patient selection.

From 2010 to 2016, 1,935 patents with stage IV breast cancer met inclusion criteria and were included in the analysis. The flowchart of patient selection is presented in Figure 1. For those patients, 52.0% (1006) underwent radiotherapy, while 48.0% (929) did not. The median survival time of the radiotherapy group and non-radiotherapy group was 56 and 45 months. No difference was found between the two groups with respect to sex, race, grade, nodal status, and the type of surgery (*P* ≥ 0.1). Patient characteristics are summarized in Table 1.

**Table 1:** Characteristics of *de novo* stage IV patients before PSM and after PSM.

**Characteristics**		**Before PSM**							**After PSM**						
		**Radiation (*****n*** **=** **1,006)**		**Non-radiation (*****n*** **=** **929)**		**Total (*****n*** **=** **1,935)**		***P*^c^**	**Radiation (*****n*** **=** **760)**		**Non-radiation (*****n*** **=** **760)**		**Total (*****n*** **=** **1,520)**		***P*^c^**
		**No**.	**52.0%**	**No**.	**48.0%**	**No**.	**100%**		**No**.	**50%**	**No**.	**50%**	**No**.	**100%**	
MST (months) (IQR)		56 (50.1–61.9)		45 (40.2–49.8)		50 (46.2–53.8)			56 (46.9–65.1)		48 (41.9–54.1)		52 (47.2–56.8)		
Age (years)	20–49	412	41	312	33.6	724	37.4	**0.001**	285	37.5	277	36.4	562	40	0.71
	50–70	497	49.4	492	53	989	51.1		475	62.5	483	63.4	958	60	
	≥70	97	9.6	125	13.5	222	11.5								
Sex	Male	19	1.9	8	0.9	27	1.4	0.083	10	1.3	8	1.1	18	1.2	0.813
	Female	987	98.1	921	99.1	1,908	98.6		750	98.7	752	98.9	1,502	98.8	
Race	White	711	70.7	654	70.4	1,365	70.5	0.98	537	70.7	541	71.2	1,078	70.9	0.659
	Black	196	19.5	187	20.1	383	19.8		153	20.1	138	18.2	291	19.1	
	Other^a^	98	9.7	87	9.4	185	9.6		69	9.1	80	10.5	149	9.8	
	Unknown	1	0.1	1	0.1	2	0.1		1	0.1	1	0.1	2	0.1	
Marital status	Married	542	53.9	458	49.3	1,000	51.7	**0.05**	388	51.1	387	50.9	775	51	0.92
	Non-married^b^	427	42.4	421	45.3	848	43.8		339	44.6	343	45.1	682	44.9	
	Unknown	37	3.7	50	5.4	87	4.5		33	4.3	30	3.9	63	4.1	
Grade	I	25	2.5	27	2.9	52	2.7	0.749	20	2.6	19	2.5	39	2.6	0.984
	II	288	28.6	250	26.9	538	27.8		212	27.9	207	27.2	419	27.6	
	III/IV	649	64.5	606	65.2	1,255	64.9		495	65.1	499	65.7	994	65.4	
	Unknown	44	4.4	46	5	90	4.7		33	4.3	35	4.6	68	4.5	
Tumor status	T1	79	7.9	87	9.4	166	8.6	**0.058**	66	8.7	70	9.2	136	8.9	0.83
	T2	355	35.3	333	35.8	688	35.6		275	36.2	263	34.6	538	35.4	
	T3	190	18.9	205	22.1	395	20.4		149	19.6	161	21.2	310	20.4	
	T4	382	38	304	32.7	686	35.5		270	35.5	266	35.0	536	35.3	
Nodal status	N0	41	4.1	46	5.0	87	4.5	0.471	35	4.6	37	4.9	72	4.7	0.971
	N1	476	47.3	421	45.3	897	46.4		355	46.7	350	46.1	705	46.4	
	N2	219	21.8	223	24	442	22.8		175	23.0	169	22.2	344	22.6	
	N3	270	26.8	239	25.7	509	26.3		195	25.7	204	26.8	399	26.3	
Breast subtype	HR+/HER2–	450	44.7	358	38.5	808	41.8	**0.052**	313	41.2	320	42.1	633	41.6	0.931
	HR+/HER2+	222	22.1	224	24.1	446	23.0		172	22.6	172	22.6	344	22.6	
	HR–/HER2+	149	14.8	152	16.4	301	15.6		117	15.4	120	15.8	237	15.6	
	HR–/HER2–	185	18.4	195	21	380	19.6		158	20.8	148	19.5	306	20.1	
Status of distant metastases	Bone-only	432	42.9	293	31.5	725	37.5	**0.001**	298	39.2	287	37.8	585	38.3	0.95
	Visceral-only	212	21.1	306	32.9	518	26.8		209	27.5	213	28	422	27.8	
	Visceral + bone	108	10.7	173	18.6	281	14.5		101	13.3	105	13.8	206	13.6	
	others	254	25.2	157	16.9	411	21.2		152	20.0	155	20.4	307	20.2	
Type of surgery	Mastectomy	766	76.1	699	75.2	1,465	75.7	0.297	569	74.9	573	75.4	1,142	75.1	0.574
	BCS	238	23.7	224	24.1	462	23.9		190	25	184	24.2	374	24.6	
	Unknown	2	0.2	6	0.6	8	0.4		1	0.1	3	0.4	4	0.3	

*PSM, perform propensity score matching; MST, Median survival time; IQR, Interquartile range; HR, Hazard Ratio; CI, confidence interval; BCSS, breast cancer-specific survival; OS, overall survival; HR, Hormone receptor (estrogen receptor and progesterone receptor); HER2, Human epidermal growth factor receptor II; TNBC, Triple-Negative breast cancer; BCS, Breast-conserving Surgery*.

a *“Other” includes American Indian/Alaskan native and Asian/Pacific Islander*.

b *“Non-married” includes divorced, separated, single (never married), unmarried or domestic partner and widowed*.

c *The P-value was calculated among all groups by the Chi-square test, and bold type indicates significance*.

### Comparison of Survival Between the Radiotherapy and Non-radiotherapy Groups

To investigate the prognostic factors, univariate and multivariate Cox proportional hazard regression models were used (Table 2). As shown in Table 2, female, non-married status, Grade II, Grade III/IV, T3, T4, N3, HR+/Her2+, HR–/Her2+, triple-negative breast cancer, and viscera + bone metastases are associated with an unfavorable BCSS or OS. Meantime, the results of multivariate analysis show that patients who received radiotherapy achieved significantly better BCSS and OS than those who did not receive radiotherapy (BCSS: Hazard Ratio, 0.737; 95% confidence interval, 0.635–0.856; *P* < 0.001; OS: Hazard Ratio, 0.733; 95% confidence interval, 0.634–0.847; *P* < 0.001, Table 2).

**Table 2:** Univariate Cox proportional hazard model and multivariate Cox proportional hazard model of breast cancer-specific survival (BCSS) and overall survival (OS) before PSM.

**Variables**		**Univariate Cox proportional hazard model**				**Multivariate Cox proportional hazard model**			
		**BCSS**		**OS**		**BCSS**		**OS**	
		**HR(95% CI)**	***P*^c^**	**HR (95% CI)**	***P*^c^**	**HR(95% CI)**	***P*^c^**	**HR (95% CI)**	***P*^c^**
Age (years)	20–49	1.00 Reference		1.00 Reference		1.00 Reference		1.00 Reference	
	50–69	1.157 (0.988–1.354)	0.07	1.19 (1.021–1.388)	**0.026**	1.057 (0.9–1.243)	0.479	1.09 (0.932–1.276)	0.28
	≥70	1.58 (1.254–1.991)	** <0.001**	1.673 (1.339–2.089)	** <0.001**	1.156 (0.91–1.468)	0.234	1.221 (0.97–1.536)	0.089
Sex	Male	1.00 Reference		1.00 Reference		1.00 Reference		1.00 Reference	
	Female	0.664 (0.391–1.126)	0.129	0.658 (0.395–1.097)	0.109	0.488 (0.283–0.840)	**0.01**	0.505 (0.299–0.854)	**0.011**
Race	White	1.00 Reference		1.00 Reference		1.00 Reference		1.00 Reference	
	Black	1.346 (1.134–1.598)	**0.001**	1.367 (1.159–1.614)	** <0.001**	1.113 (0.931–1.332)	0.241	1.146 (0.964–1.362)	0.123
	Other**^a^**	0.77 (0.586–1.01)	0.059	0.746 (0.571–0.974)	**0.031**	0.828 (0.63–1.089)	0.177	0.805 (0.615–1.053)	0.114
	Unknown	0 (0–4.94E + 76)	0.933	0 (0+7.79E + 74)	0.931	0.002 (0–5.03E + 57)	0.929	0.002 (0–3.20E + 55)	0.927
Marital status	Married	1.00 Reference		1.00 Reference		1.00 Reference		1.00 Reference	
	Non-married**^b^**	1.316 (1.136–1.526)	** <0.001**	1.356 (1.176–1.565)	** <0.001**	1.245 (1.065–1.456)	**0.006**	1.272 (1.093–1.48)	**0.002**
	Unknown	1.23 (0.883–1.712)	0.221	1.197 (0.864–0.66)	0.28	1.186 (0.85–1.658)	0.315	1.155 (0.831–1.606)	0.392
Grade	I	1.00 Reference		1.00 Reference		1.00 Reference		1.00 Reference	
	II	1.758 (0.928–3.334)	0.084	1.39 (0.806–2.398)	0.236	1.948 (1.023–3.71)	**0.042**	1.534 (0.885–2.657)	0.127
	III/IV	3.091 (1.653–5.778)	** <0.001**	2.32 (1.365–3.943)	**0.002**	2.71 (1.435–5.119)	**0.002**	2.039 (1.186–3.503)	**0.01**
	Unknown	2.529 (1.252–5.109)	**0.01**	2.025 (1.099–3.73)	**0.024**	2.2 (1.081–4.477)	**0.03**	1.768 (0.952–3.282)	0.071
Tumor status	T1	1.00 Reference		1.00 Reference		1.00 Reference		1.00 Reference	
	T2	1.407 (1.017–1.946)	**0.039**	1.447 (1.054–1.985)	**0.022**	1.312 (0.947–1.819)	0.102	1.363 (0.992–1.874)	0.056
	T3	1.765 (1.262–2.47)	**0.001**	1.792 (1.291–2.487)	** <0.001**	1.398 (0.993–1.968)	0.055	1.447 (1.036–2.02)	**0.03**
	T4	2.114 (1.535–2.911)	** <0.001**	2.156 (1.577–2.947)	** <0.001**	1.595 (1.147–2.219)	**0.006**	1.64 (1.188–2.264)	**0.003**
Nodal status	N0	1.00 Reference		1.00 Reference		1.00 Reference		1.00 Reference	
	N1	0.908 (0.643–1.282)	0.583	0.938 (0.668–1.317)	0.711	1.176 (0.824–1.677)	0.372	1.208 (0.852–1.713)	0.289
	N2	1.038 (0.726–1.485)	0.839	1.079 (0.759–1.535)	0.671	1.297 (0.896–1.878)	0.169	1.347 (0.936–1.938)	0.109
	N3	1.097 (0.77–1.563)	0.61	1.158 (0.818–1.641)	0.408	1.398 (0.968–2.019)	0.074	1.481 (1.033–2.123)	**0.003**
Breast subtype	HR+/HER2–	1.00 Reference		1.00 Reference		1.00 Reference		1.00 Reference	
	HR+/HER2+	0.587 (0.471–0.733)	** <0.001**	0.578 (0.467–0.715)	** <0.001**	0.506 (0.403–0.635)	** <0.001**	0.501 (0.403–0.624)	** <0.001**
	HR–/HER2+	0.803 (0.633–1.019)	0.071	0.766 (0.616–0.978)	**0.032**	0.685 (0.535–0.876)	**0.003**	0.673 (0.53–0.854)	**0.001**
	HR–/HER2–	2.938 (2.48–3.479)	** <0.001**	2.8 (2.376–3.3)	** <0.001**	2.59 (2.145–3.128)	** <0.001**	2.486 (2.069–2.987)	** <0.001**
Status of distant metastases	Bone-only	1.00 Reference		1.00 Reference		1.00 Reference		1.00 Reference	
	Visceral-only	1.478 (1.229–1.778)	** <0.001**	1.479 (1.237–1.768)	** <0.001**	1.188 (0.977–1.446)	0.085	1.206 (0.998–1.458)	0.53
	Visceral + bone	1.925 (1.565–2.368)	** <0.001**	1.881 (1.537–2.302)	** <0.001**	1.859 (1.499–2.304)	** <0.001**	1.821 (1.477–2.245)	** <0.001**
	Unknown	1.129 (0.919–1.388)	0.248	1.143 (0.937–1.394)	0.189	0.884 (0.713–1.097)	0.263	0.908 (0.738–1.118)	0.363
Type of surgery	BCS	1.00 Reference		1.00 Reference		1.00 Reference		1.00 Reference	
	Mastectomy	1.165 (0.98–1.386)	0.084	1.154 (0.976–1.365)	0.094	1.125 (0.939–1.348)	0.203	1.106 (0.929–1.318)	0.259
	Unknown	0.987 (0.315–3.092)	0.982	1.232 (0.457–3.319)	0.68	1.233 (0.386–3.944)	0.724	1.509 (0.548–4.156)	0.427
Radiation	No	1.00 Reference		1.00 Reference		1.00 Reference		1.00 Reference	
	Yes	0.733 (0.635–0.847)	** <0.001**	0.729 (0.634–0.838)	** <0.001**	0.737 (0.635–0.856)	** <0.001**	0.733 (0.634–0.847)	** <0.001**

*PSM, perform propensity score matching; HR, Hazard Ratio; CI, confidence interval; BCSS, breast cancer-specific survival; OS, overall survival; HR, Hormone receptor (estrogen receptor and progesterone receptor); HER2, Human epidermal growth factor receptor II; TNBC, Triple-Negative breast cancer; BCS, Breast-conserving Surgery*.

a *“Other” includes American Indian/Alaskan native and Asian/Pacific Islander*.

b *“Non-married” includes divorced, separated, single (never married), unmarried or domestic partner and widowed*.

c *The P-value was adjusted by the univariate Cox proportional hazard regression model including all factors, and bold type indicates significance*.

### Survival Estimates in Matched Groups

Propensity score matching (1:1) between the radiation group and non-radiation group was performed with consideration of all variables that may affect the outcome of breast cancer (age, sex, race, marriage, grade, tumor status, N status, the type of surgery, subtype, and metastatic sites) (Table 3). After PSM, a total of 1,520 patients with 760 patients in each group were enrolled in this analysis. There were no significant differences for any variables.

**Table 3:** Univariate Cox proportional hazard model and multivariate Cox proportional hazard model of breast cancer-specific survival (BCSS) and overall survival (OS) after PSM.

**Variables**		**Univariate Cox proportional hazard model**				**Multivariate Cox proportional hazard model**			
		**BCSS**		**OS**		**BCSS**		**OS**	
		**HR(95% CI)**	***P*^c^**	**HR (95% CI)**	***P*^c^**	**HR(95% CI)**	***P*^c^**	**HR (95% CI)**	***P*^c^**
Age (years)	20–49	1.00 Reference		1.00 Reference		1.00 Reference		1.00 Reference	
	≥50	1.194 (1.004–1.419)	**0.044**	1.229 (1.039–1.455)	**0.016**	1.136 (0.951–1.358)	0.161	1.167 (0.98–1.388)	0.083
Sex	Male	1.00 Reference		1.00 Reference		1.00 Reference		1.00 Reference	
	Female	0.691 (0.344–1.388)	0.299	0.649 (0.336–1.255)	0.199	0.506 (0.248–1.033)	0.061	0.503 (0.256–0.986)	**0.046**
Race	White	1.00 Reference		1.00 Reference		1.00 Reference		1.00 Reference	
	Black	1.365 (1.121–1.662)	**0.002**	1.36 (1.124–1.646)	**0.002**	1.173 (0.954–1.441)	0.13	1.181 (0.967–1.442)	0.103
	Other**^a^**	0.808 (0.595–1.097)	0.171	0.773 (0.572–1.045)	0.094	0.857 (0.63–1.166)	0.325	0.813 (0.6–1.102)	0.182
	Unknown	0 (0–7.06E + 76)	0.933	0 (0–1.49E + 75)	0.931	0.002 (0–3.44E + 57)	0.93	0.002 (0–2.41E + 55)	0.928
Marital status	Married	1.00 Reference		1.00 Reference		1.00 Reference		1.00 Reference	
	Non-married**^b^**	1.303 (1.102–1.541)	**0.002**	1.325 (1.126–1.559)	**0.001**	1.275 (1.07–1.52)	**0.007**	1.29 (1.087–1.53)	**0.003**
	Unknown	1.082 (0.718–1.631)	0.705	1.066 (0.713–1.594)	0.754	1.146 (0.757–1.735)	0.518	1.132 (0.754–1.698)	0.55
Grade	I	1.00 Reference		1.00 Reference		1.00 Reference		1.00 Reference	
	II	1.557 (0.76–3.188)	0.226	1.263 (0.682–2.338)	0.457	1.553 (0.754–3.2)	0.233	1.265 (0.678–2.357)	0.46
	III/IV	2.779 (1.381–5.595)	**0.004**	2.106 (1.157–3.832)	**0.015**	2.188 (1.072–4.464)	**0.031**	1.685 (0.913–3.111)	0.095
	Unknown	2.036 (0.914–4.533)	0.082	1.674 (0.83–3.375)	0.15	1.736 (0.771–3.907)	0.183	1.45 (0.711–2.958)	0.307
Tumor status	T1	1.00 Reference		1.00 Reference		1.00 Reference		1.00 Reference	
	T2	1.337 (0.936–1.912)	0.111	1.434 (1.005–2.047)	**0.047**	1.264 (0.882–1.813)	0.202	1.381 (0.965–1.976)	0.078
	T3	1.76 (1.217–2.545)	**0.003**	1.856 (1.286–2.679)	**0.001**	1.463 (1.003–2.135)	**0.048**	1.595 (1.096–2.322)	0.015
	T4	2.005 (1.41–2.852)	** <0.001**	2.146 (1.511–3.047)	** <0.001**	1.463 (1.016–2.106)	**0.041**	1.596 (1.111–2.291)	0.011
Nodal status	N0	1.00 Reference		1.00 Reference		1.00 Reference		1.00 Reference	
	N1	0.967 (0.654–1.429)	0.866	0.989 (0.674–1.451)	0.954	1.291 (0.863–1.931)	0.213	1.319 (0.889–1.958)	0.169
	N2	1.089 (0.725–1.633)	0.682	1.105 (0.742–1.646)	0.622	1.487 (0.976–2.266)	0.065	1.498 (0.991–2.264)	0.055
	N3	1.177 (0.787–1.76)	0.427	1.234 (0.832–1.83)	0.295	1.575 (1.036–2.396)	**0.034**	1.651 (1.095–2.488)	**0.017**
Breast subtype	HR+/HER2-	1.00 Reference		1.00 Reference		1.00 Reference		1.00 Reference	
	HR+/HER2+	0.547 (0.422–0.71)	** <0.001**	0.528 (0.411–0.679)	** <0.001**	0.483 (0.37–0.63)	** <0.001**	0.471 (0.365–0.61)	** <0.001**
	HR–/HER2+	0.856 (0.656–1.116)	0.251	0.797 (0.614–1.033)	0.086	0.749 (0.57–0.985)	**0.039**	0.71 (0.542–0.929)	**0.012**
	HR–/HER2–	2.914 (2.405–3.53)	** <0.001**	2.719 (2.257–3.276)	** <0.001**	2.746 (2.217–3.402)	** <0.001**	2.589 (2.102–3.188)	** <0.001**
Status of distant metastases	Bone–only	1.00 Reference		1.00 Reference		1.00 Reference		1.00 Reference	
	Visceral-only	1.342 (1.088–1.654)	**0.006**	1.308 (1.067–1.604)	**0.01**	1.157 (0.929–1.44)	0.192	1.155 (0.933–1.43)	0.185
	Visceral + bone	1.969 (1.559–2.488)	** <0.001**	1.927 (1.535–2.42)	** <0.001**	1.956 (1.542–2.505)	** <0.001**	1.941 (1.532–2.459)	** <0.001**
	Unknown	1.147 (0.905–1.453)	0.256	1.169 (0.932–1.467)	0.177	0.847 (0.661–1.085)	0.188	0.883 (0.696–1.121)	0.306
Type of surgery	BCS	1.00 Reference		1.00 Reference		1.00 Reference		1.00 Reference	
	Mastectomy	1.157 (0.951–1.407)	0.145	1.167 (0.965–1.412)	0.111	1.128 (0.919–1.383)	0.249	1.122 (0.919–1.369)	0.257
	Unknown	1.593 (0.394–6.441)	0.514	1.523 (0.377–6.157)	0.555	3.609 (0.867–15.019)	0.078	3.441 (0.829–14.285)	0.089
Radiation	No	1.00 Reference		1.00 Reference		1.00 Reference		1.00 Reference	
	Yes	0.776 (0.658–0.915)	**0.002**	0.787 (0.671–0.923)	**0.003**	0.697 (0.59–0.823)	** <0.001**	0.707 (0.601–0.831)	** <0.001**

*PSM, perform propensity score matching; HR, Hazard Ratio; CI, confidence interval; BCSS, breast cancer-specific survival; OS, overall survival; HR, Hormone receptor (estrogen receptor and progesterone receptor); HER2, Human epidermal growth factor receptor II; TNBC, Triple-Negative breast cancer; BCS, Breast-conserving Surgery*.

a *“Other” includes American Indian/Alaskan native and Asian/Pacific Islander*.

b *“Non-married” includes divorced, separated, single (never married), unmarried or domestic partner and widowed*.

c *The P-value was adjusted by the univariate Cox proportional hazard regression model including all factors, and bold type indicates significance*.

**Figure 2:**
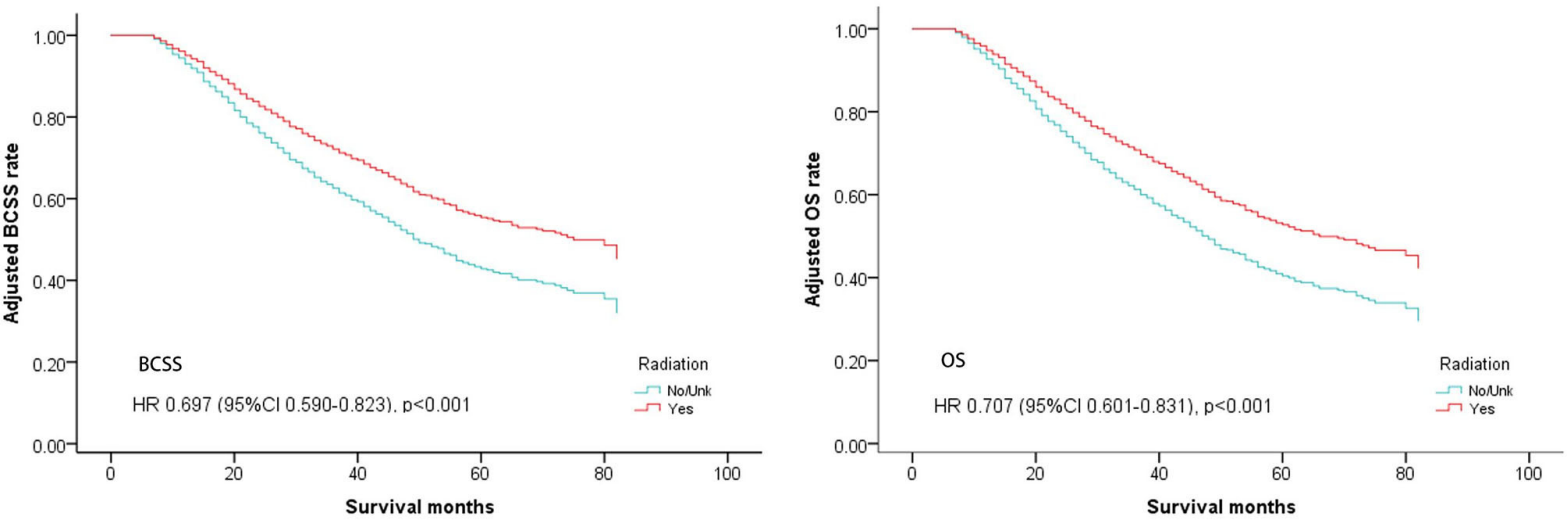
Survival curves of BCSS and OS for the general population after PSM; radiotherapy vs. non-radiotherapy (adjusted by age, marital, race, sex, grade, tumor, node, surgery, distant, subtype).

Multivariate analysis for BCSS and OS after PSM found that, female, non-married status, Grade III/IV, T3, T4, N3,HR+/Her2+, HR–/Her2+, triple-negative breast cancer, and viscera + bone metastases were associated with a more unfavorable prognosis which is consistent with the results of multivariate analysis before PSM. The radiation group also had significantly better outcomes for both BCSS and OS (BCSS: Hazard Ratio, 0.697; 95% confidence interval, 0.59–0.823; *P* < 0.001; OS: Hazard Ratio, 0.707; 95% confidence interval, 0.601–0.831; *P* < 0.001; Table 3, Figure 2).

### Subgroup Analysis According to Age, Breast Subtype, Status of Distant Metastasis, and Type of Surgery

We further investigated the impact of postoperative radiotherapy on BCSS and OS in different subgroups.

**Figure 3:**
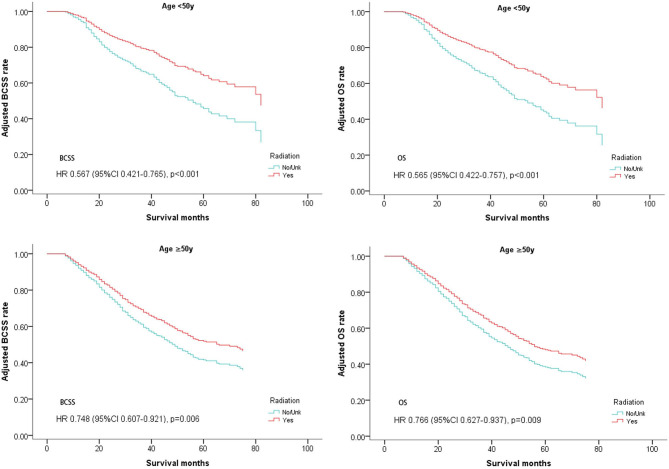
Survival curves of BCSS and OS for the general population stratified by age after PSM; radiotherapy vs. non-radiotherapy.

A subgroup analysis was first performed in which patients were stratified by age. As shown in Table 4, clinical benefits from postoperative radiotherapy could be seen in the overall population regardless of age. (Age <50, BCSS: Hazard Ratio, 0.567; 95% confidence interval, 0.421–0.765; *P* < 0.001; OS: Hazard Ratio, 0.565; 95% confidence interval, 0.422–0.757; *P* < 0.001; Age ≥ 50, BCSS: Hazard Ratio, 0.748; 95% confidence interval, 0.607–0.921; *P* = 0.006; OS: Hazard Ratio, 0.766; 95% confidence interval, 0.627–0.937; *P* = 0.009; Table 4, Figure 3).

**Table 4:** Multivariate Cox proportional hazard regression model of breast cancer-specific survival (BCSS) and overall survival (OS) for the radiotherapy and non-radiotherapy groups, stratified according to clinical variables after PSM.

**Variables^b^**	**Radiotherapy vs. non-radiotherapy^a^**			
	**BCSS**		**OS**	
	**HR (95% CI)**	***P*^c^**	**HR (95% CI)**	***P*^c^**
**Age**				
<50 y	0.567 (0.421–0.765)	** <0.001**	0.565 (0.422–0.757)	** <0.001**
≥50 y	0.748 (0.607–0.921)	**0.006**	0.766 (0.627–0.937)	**0.009**
**Subtype**				
HR+/HER2–	0.675 (0.516–0.883)	**0.004**	0.702 (0.544–0.906)	**0.007**
HR+/HER2+	0.939 (0.574–1.537)	0.803	0.986 (0.614–1.586)	0.955
HR–/HER2+	0.778 (0.475–1.273)	0.317	0.739 (0.455–1.202)	0.223
HR–/HER2–	0.586 (0.435–0.788)	** <0.001**	0.582 (0.434–0.781)	** <0.001**
**Status of distant metastases**				
Bone-only	0.689 (0.511–0.928)	**0.014**	0.749 (0.561–0.999)	**0.049**
Visceral-only	0.735 (0.529–1.022)	0.067	0.719 (0.52–0.993)	**0.046**
Visceral + bone	0.753 (0.503–1.127)	0.168	0.77 (0.519–1.142)	0.194
Unknown	0.495 (0.33–0.743)	**0.001**	0.477 (0.324–0.703)	** <0.001**
**Type of surgery**				
BCS	0.512 (0.352–0.745)	** <0.001**	0.51 (0.353–0.735)	** <0.001**
Mastectomy	0.725 (0.602–0.881)	**0.001**	0.741 (0.617–0.892)	**0.001**

*PSM, perform propensity score matching; HR, Hazard Ratio; CI, confidence interval; BCSS, breast cancer-specific survival; OS, overall survival; HR, Hormone receptor (estrogen receptor and progesterone receptor); HER2, Human epidermal growth factor receptor II; TNBC, Triple-Negative breast cancer; BCS, Breast-conserving Surgery*.

a *Using non-radiotherapy as a reference*.

b *Adjusted using a multivariate Cox proportional hazard regression model, including age, race, marital status, grade, tumor size, lymph node status, breast subtype, surgery and metastasis*.

c *Bold type indicates significance*.

**Figure 4:**
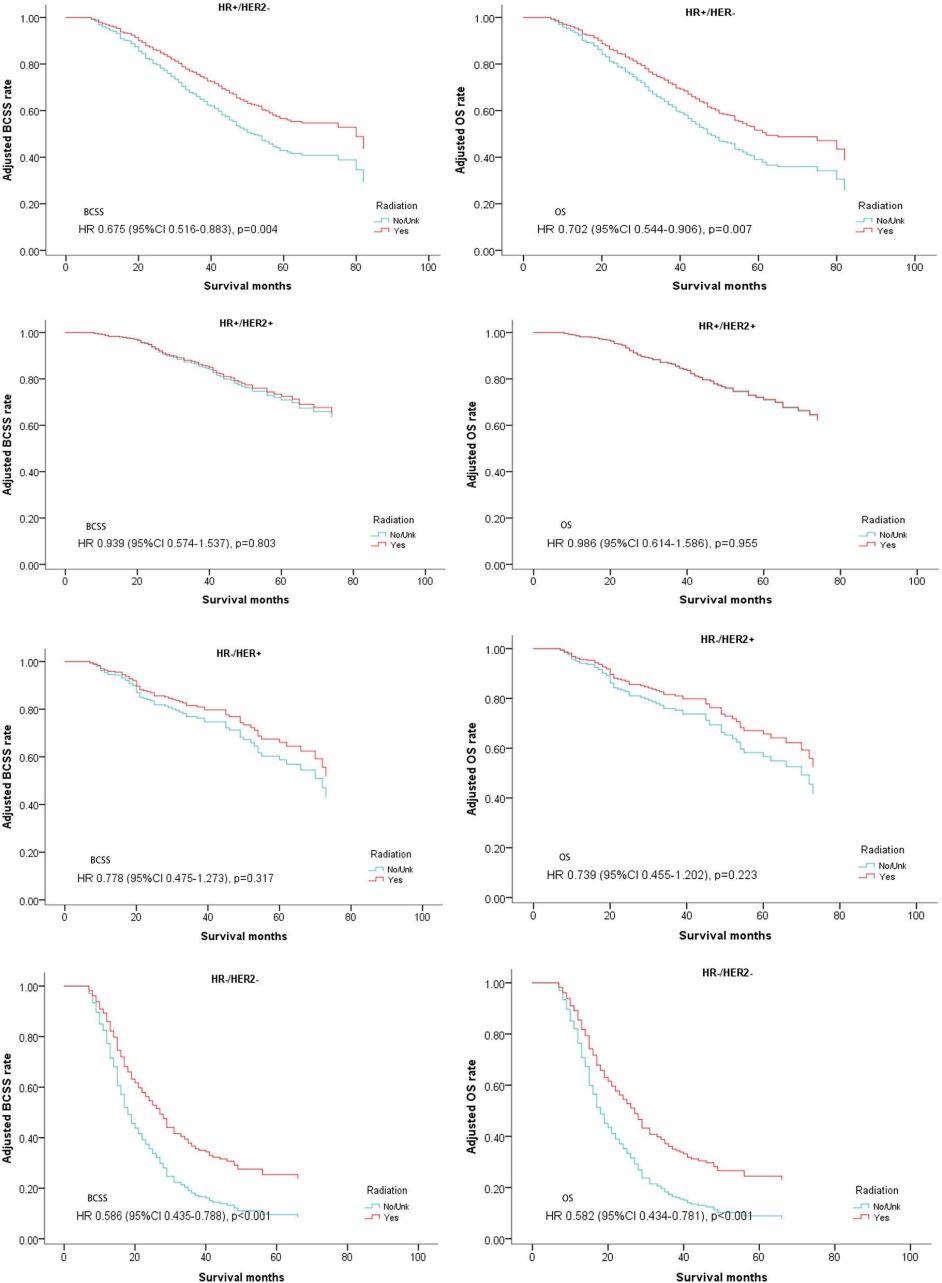
Survival curves of BCSS and OS for the general population stratified by subtypes after PSM; radiotherapy vs. non-radiotherapy.

These patients were then divided into four groups according to the expression of hormone receptors and Her2 receptor (subtypes: HR+/Her2–, HR+/Her2+, HR–/Her2+, HR–/Her2–). Multivariate analysis for BCSS and OS demonstrated that luminal subtype breast cancer (HR+/Her2–) and triple negative breast cancer (HR–/Her2–) may benefit from postoperative radiotherapy (HR+/Her2–; BCSS: Hazard Ratio, 0.675; 95% confidence interval, 0.516–0.883; *P* = 0.004; OS: Hazard Ratio, 0.702; 95% confidence interval, 0.544–0.906; *P* = 0.007; TNBC: BCSS: Hazard Ratio, 0.586; 95% confidence interval, 0.435–0.788; *P* < 0.001; OS: Hazard Ratio, 0.582; 95% confidence interval, 0.434–0.781; *P* < 0.001; Table 4, Figure 4).

**Figure 5:**
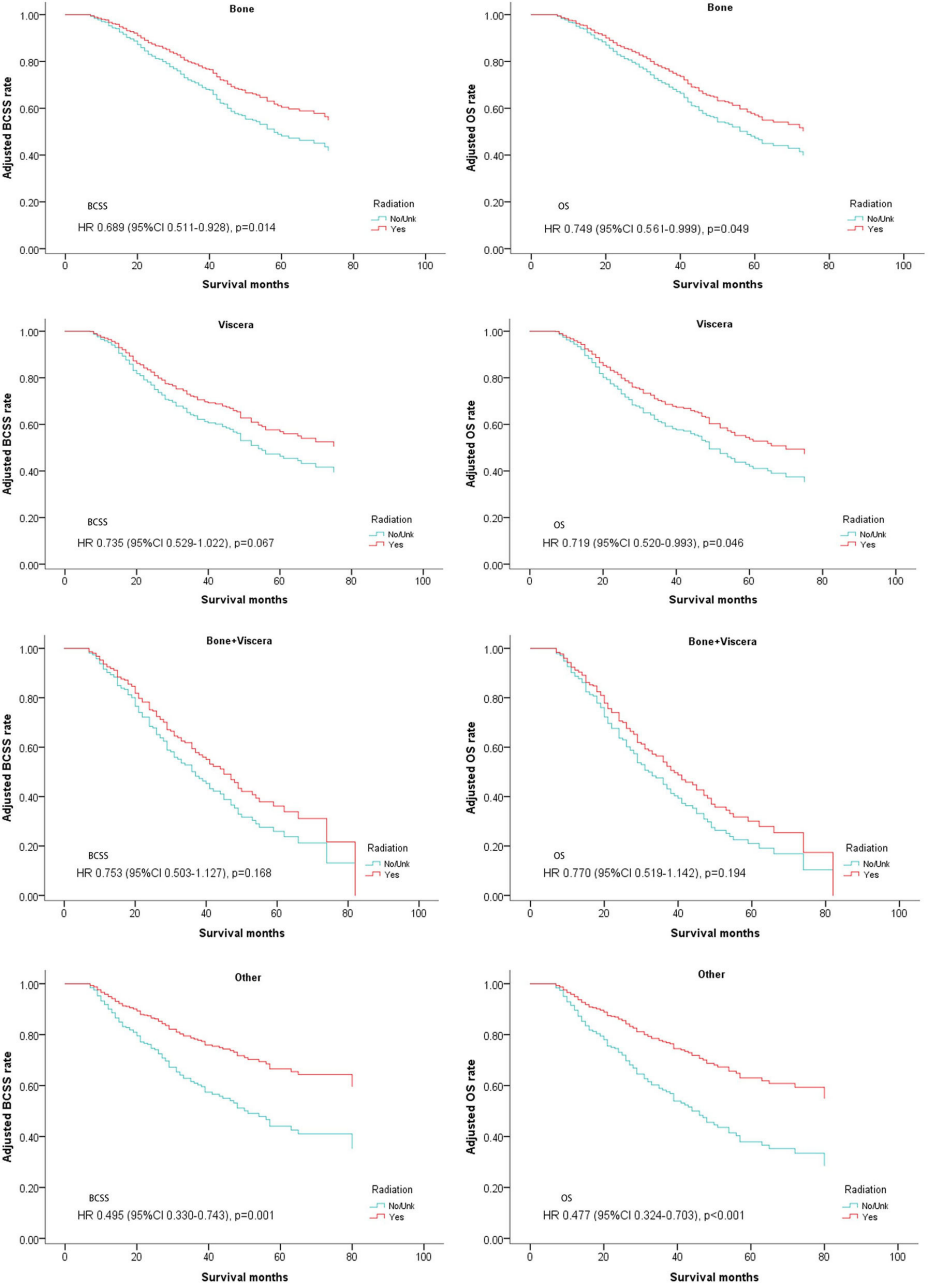
Survival curves of BCSS and OS for the general population stratified by metastasis sites after PSM; radiotherapy vs. non-radiotherapy.

Since metastatic sites are always associated with different outcomes and affect the treatment choice, these patients were stratified by different metastasis location (bone metastasis, viscera metastasis, viscera+bone metastases, and other metastasis). A significantly better OS after radiotherapy was found in nearly all the subgroups except for the patients with both bone and viscera metastases. However, on multivariate analysis for BCSS, radiotherapy provided favorable prognosis in bone-only metastasis and other metastasis which mainly indicated distant lymph node metastases (Table 4, Figure 5).

**Figure 6:**
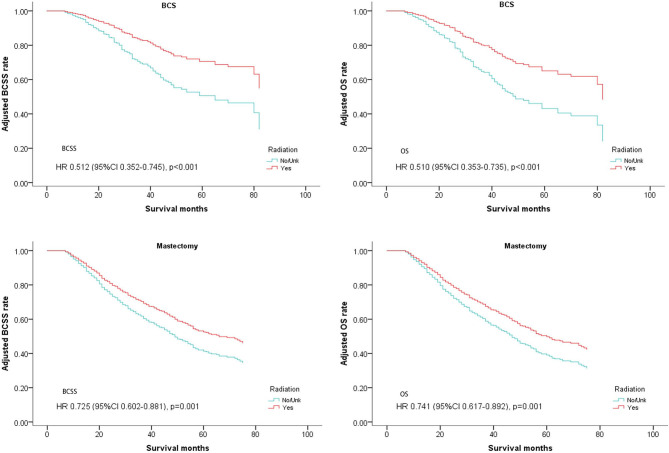
Survival curves of BCSS and OS for the general population stratified by operation methods after PSM; radiotherapy vs. non-radiotherapy.

The patients who received BCS and mastectomy were divided into two subgroups. Radiotherapy was correlated with a significantly better prognosis regardless of whether it was used after BCS or mastectomy (BCS: BCSS: Hazard Ratio, 0.512; 95% confidence interval, 0.352–0.745; *P* < 0.001; OS: Hazard Ratio, 0.51; 95% confidence interval, 0.353–0.735; *P* < 0.001; Mastectomy: BCSS: Hazard Ratio, 0.725; 95% confidence interval, 0.602–0.881; *P* = 0.001; OS: Hazard Ratio, 0.741; 95% confidence interval, 0.617–0.892; *P* = 0.001; Table 4, Figure 6).

## Discussion

In recent years several prospective clinical trials have presented negative results after performing an operation for dnMBC patients (3, 6). Those patients who achieved tumor remission from systemic treatment do not seem to survive longer from additional local treatments. Although a positive result was not observed in the general population, we could not assign this conclusion to every single patient as individual disease characteristics are quite different from each other in dnMBC. Otherwise, LRT could decrease local recurrence, as shown in those studies. In fact, in our clinical practice, we always recommended LRT to those patients who have longer life expectancies. Retrospective studies have proven the clinical value of LRT in this specific population. Our study aimed to evaluate radiotherapy after surgery, a kind of aggressive local treatments in dnMBC. Despite the role of radiation in stage IV breast cancer patients still being debated, it has been frequently used in dnMBC and performed not just as palliative treatment but also as enhanced local treatment to improve OS. We investigated whether aggressive local treatment (radiotherapy plus surgery) leads to better outcomes when compared with surgery alone. In our study, we only evaluated the efficacy of postoperative radiotherapy in those patients who were sensitive to systemic therapy and received surgery for primary tumor. We demonstrated that adding radiation would increase the BCSS and OS in the entire cohort of dnMBC patients. Radiotherapy following surgery is a routine recommendation to node positive or local advanced early breast cancer patients since it can reduce local recurrence and even improve overall survival of the patients (21, 22). Enhancing local control may be helpful to reduce the tumor burden, eradicate local micro-metastatic disease, and increase the effectiveness of systemic treatments in dnMBC. As well, it may limit additional reseeding of the tumor. However, recent studies discovered that radiation might have broader systemic effects. There is a theory that radiotherapy induced cancer necrosis and any endogenous cancer specific antigens exposed may lead to innate immune response, called an abscopal effect, and result in distant cancer remission (23–26). This will strengthen the theoretical foundation for performing radiotherapy for dnMBC patients. Yet it must be admitted that there are limits to our study. Since the SEER database does not provide the information on the radiation sites, we could not be certain that all the patients in the radiation group received the radiotherapy to primary areas, although we did try to exclude as many patients as possible who had received radiotherapy for metastatic sites. But this limitation would not bring too much uncertainty to our conclusion. Aggressive local treatment by adding radiotherapy to surgery, whether to local regional areas or metastatic areas, would bring better outcomes to at least a group of dnMBC patients.

By performing subgroup analyses we tried to determine who would be more likely to benefit from postoperative radiotherapy. As shown in the stratified analysis of breast tumor subtype, luminal breast (HR+/HER2–) cancer patients could benefit from radiotherapy. This result was in accordance with results from MF07-01 that also showed better results of local regional treatment (LRT) in ER/PR+ and/or HER2– subgroup (5). Longer survival time of these patients may contribute to enhance the therapeutic effects of additional local treatment. Therefore, the HR and HER2 status could be good candidates for selecting postoperative radiotherapy. However, in contrast to previous studies, the BCSS and OS of metastatic triple negative breast cancer (mTNBC) was also significantly raised (5, 6). Although the mechanism is ambiguous, several results have shown potential correlations between radiotherapy and the improvement of the mTNBC patients' prognosis. A related report indicated that lack of a functional BRCA-1 gene may increase the sensitivity of cells to radiation. Therefore, we believe that one of the reasons why radiotherapy works in TNBC might relate to BRCA-1 gene mutations as such gene mutations usually occur in TNBC (27, 28). The disability of the gene acts as a trigger which increases the effect of radiation on cancer cells and improves patient survival. Another concept is that radiation may stimulate the immune system, as mentioned above. The TNBC tumors are considered the most immunogenetic subtype, mainly attributed to the high number of tumor-infiltrating lymphocytes (TILs) (25). Based on high TIL numbers, radiation could also increase the percentage of antigen-experienced T cells and effector memory T cells (29, 30). Both of these mechanisms may enhance the immune response in mTNBC patients. Once the immune system is activated, increasing concentrations of molecules associated with a pro-inflammatory immune responses, such as tumor necrosis factor (TNF), can promote antigen presentation, and stimulate the T-cells and lead to activation of the corresponding immune response in mTNBC patients and the killing of tumor cells (31). These effects make radiotherapy a modality potentially synergistic with the immune system and may result in a survival advantage for mTNBC patents. Additionally, recent studies showed that low-dose fractionated radiotherapy could up-regulate the expression of PD-L1 on tumor cells which make it possible to combine radiation therapy and PD-1/PD-L1 signaling blockade to generate effective anti-tumor immunity and achieve long-term tumor control (32, 33). There are several ongoing clinical trials investigating the use of combined immunoradiotherapy in treatment of TNBC (NCT02303366, NCT02730130, NCT02499367). All of these factors may change the role of radiotherapy and enhance its clinical value in the future.

There are clinical results that show the irreplaceable role of radiation after BCS to reduce local recurrence and prolong survival (34). Adding radiation after mastectomy also leads to better survival in node positive patients (22). Therefore, we continue to investigate the effects of postoperative radiotherapy according to different operation methods, BCS and mastectomy. In a previous study radiation was demonstrated to be effective after BCS for dnMBC patients, consistent with our findings (13). However, Huang et al. and Ly et al. drew opposite conclusions in their studies of the effects of post mastectomy radiation (14, 35). Ly concluded that surgery and radiotherapy were associated with a significant survival advantage in the general population, but there was no difference in survival among the subgroup receiving mastectomy. However, Huang recommended that the patients with stage IV breast cancer should be given intensive local treatment including radiotherapy after chemotherapy and mastectomy. The different inclusion criteria used by the two studies may have led to different results. Not considering the effects of systemic therapy in Ly's study is an error. In our study, we excluded the patients who did not receive chemotherapy, consistent with Huang's study, and supports radiotherapy improving the survival rate in dnMBC patients regardless of surgical type. We think that elimination of micro-metastasis by radiotherapy not removed by surgery, such as the metastasis in subcutaneous lymphatics or in supraclavicular lymph nodes, can lead to a better outcome. Obviously, compared with early breast cancer, metastatic breast cancer has more opportunities for residual micro-metastasis.

The subgroup analysis from MF0701indicated that enhanced local treatment might be associated with a better prognosis for patients with bone-only metastases (5). Patients with a lower metastasis burden have a better chance to achieve a better outcome from surgery and local radiation. In our study, those patients were stratified according to metastasis burden (bone metastasis, viscera metastasis, viscera + bone metastasis, and other metastasis). Since other metastasis are always refer to as lymph node metastasis in the SEER database, this group of patients also have a low tumor burden. Both groups with bone-only metastasis and other metastasis (most of them are lymph node metastasis) showed better BCSS results in the radiation treatment groups. Three other groups, excepting simultaneous bone and viscera metastasis, had better OS. This observation supported metastasis burden as an important factor to consider for the treatment decisions. Patients with a low metastasis burden are a suitable population for postoperative radiotherapy.

Finally, we did not find that age was an influential factor for the efficacy of radiation. Patients, whether younger than 50 or older than 50, can benefit from postoperative radiotherapy.

In our study, we included a large number of participants to evaluate the value of postoperative radiotherapy in dnMBC by using the SEER database. By excluding the patients who did not received chemotherapy and surgery, who has brain metastasis, who did not have lymph node involved, and performing PSM, we reduced the selection bias as much as possible to make our conclusion more reliable. We believe our finding can provide a reference for clinical practice. However, we admit there are several inevitable limitations. As we have mentioned before, since SEER does not provide the information about radiation sites, we could not confirmed that everyone including in our study received local regional radiation. Nevertheless, whether radiotherapy is aimed at the primary sites or metastatic sites would not change the facts that radiotherapy, as an additional local treatment could benefit dnMBC patients by enhancing local control and promoting an abscopal effect. Another limitation is due to the SEER database itself, the information about the details of endocrine therapy, targeted therapy, chemotherapy, and immune therapy are unavailable.

## Conclusion

In our study, we demonstrated that post-operative radiotherapy, whether combined with BCS or mastectomy, can bring additional benefit to dnMBC patients, especially in HR+/HER2–, triple negative breast cancer, and patients with a low metastatic burden.

## Data Availability Statement

The original contributions presented in the study are included in the article/supplementary material, further inquiries can be directed to the corresponding author/s.

## Ethics Statement

Since the data from SEER database was reported by each state of the United States and we did not involve any information from individual patients, no informed patient consent was required. However, the SEER 1973–2016 Research Data File has been established a Data-Use Agreement.

## Author Contributions

JZ: formal analysis, investigation, visualization, writing–original draft and writing - review and editing. SL: formal analysis, data curation, methodology, software, writing - review and editing. ZQ: formal analysis, data curation, methodology, supervision, validation, writing - review and editing. YL: data curation. CS: conceptualization, funding acquisition, project administration, resources, supervision, writing - review and editing. All authors read and approved the final manuscript.

## Conflict of Interest

The authors declare that the research was conducted in the absence of any commercial or financial relationships that could be construed as a potential conflict of interest.
